# Self-Radiopaque Markers Guiding Physician-Modified Fenestration (S-Fenestration) in Aortic Arch Endovascular Repair

**DOI:** 10.3389/fcvm.2021.713301

**Published:** 2021-08-20

**Authors:** Xin Li, Chang Shu, Quanming Li, Hao He, Ming Li, Lunchang Wang, Jiehua Li, Dingxiao Liu, Mingyuan Du

**Affiliations:** ^1^Department of Vascular Surgery, The Second Xiangya Hospital, Central South University, Changsha, China; ^2^The Institute of Vascular Diseases, Central South University, Changsha, China; ^3^Center of Vascular Surgery, State Key Laboratory of Cardiovascular Diseases, Fuwai Hospital, National Center for Cardiovascular Diseases, Chinese Academy of Medical Science and Peking Union Medical College, Beijing, China

**Keywords:** aortic arch pathologies, TEVAR, physician modified fenestration, stent-graft, radiopaque marker, branch stenting

## Abstract

**Backgrounds and Objectives:** Thoracic endovascular aortic repair (TEVAR) has currently become the “first-line choice” for descending aortic pathologies. For pathologies located at the aortic arch, TEVAR with physician-modified fenestration (PMF) has been gained popularity as an alternative choice. However, stent fenestration is an experience-dependent technique and comes with possible adverse events such as misalignment. This study aims to introduce the self-radiopaque PMF (SF), which uses the radiopaque marker as a guiding indicator.

**Methods:** This is a single-center retrospective study of 125 patients who underwent the SF-TEVAR in Second Xiangya Hospital from December 2015 to December 2020. Data include basic clinical information and technique records of SF-TEVAR with follow-up results.

**Results:** According to the SF-TEVAR protocol, we have performed the procedures on 125 patients and obtained an instant success rate of 98.4%. A total of 140 aortic stent-grafts and 44 bridging stents have been implanted in this study. The operation time is 64.6 ± 19.3 min, X-ray exposure time (from first digital subtraction angiography (DSA) to last DSA) is 25.6 ± 14.3 min, and contrast volume is 82.2 ± 22.6 ml. The success rate of PMF alignment is 98.4%. One bailout stent-graft was implanted into the left subclavian artery (LSA) by the chimney technique (0.8%). One fenestration was successfully and immediately corrected after misalignment (0.8%). Large simultaneous fenestration was performed in six patients (4.8%) for the left common carotid artery (LCCA) and LSA and in two patients (1.6%) for IA, LCCA, and LSA. One hundred twenty-two out of 125 patients' LSAs have been kept patent by the technique during the follow-up. The bridging stent group consists of 44 patients who received LSA stents, while the non-bridging stent group includes the other 81 patients. Type I endoleak has occurred in seven patients (5.6%) 1 week after the procedure. During follow-up (23 ± 18 months), survival rate is 95.7% and branch artery patent rate is 97.4%.

**Conclusions:** The SF-TEVAR technique, which utilizes the radiopaque marker in stent-graft as an indication for PMF in TEVAR, seems a likely safe, effective, and efficient procedure that brings acceptable survival rate and branch artery patency rate. SF-TEVAR serves as a progressive alternative method to keep the branch artery patent in aortic arch endovascular reconstruction.

## Introductions

With the rapid development of thoracic endovascular aortic repair (TEVAR) technique and device, the indications of TEVAR are assumed to expand to aortic arch (1, 2). A series of endovascular techniques have gradually been applied for reconstruction of the aortic arch, including fenestration techniques, customized stent-grafts, and chimney techniques with “skirt stent-graft” device that is specially designed for chimney-TEVAR (2–7). When designing the clinical strategies toward aortic arch pathologies, the optimal goal is considered to repair the lesion while keeping the branch arteries patent. To date, literatures have already reported several physician-modified fenestration (PMF) methods for aortic lesions at various locations, even including the ascending aorta (8–11). However, it still raises concern to physicians as to the risks such as misalignment of fenestration, proximal endoleak, and bail-out chimney stenting. Herein, we introduce a novel method that takes advantage of radiopaque markers in stent-graft itself to guide the fenestration and locate branch arteries. We therefore named this method as S-Fenestration technique for self-radiopaque PMF. Due to its advantages as less operating/anesthetic time, easier branch artery stenting procedure, and more accurate fenestration alignment, we suppose that the self-radiopaque PMF (SF) technique can probably work as an improved technique for a better mini-invasive experience, especially for the aged patients.

## Materials and Methods

This is a retrospectively study of 125 patients who underwent the SF-TEVAR in Second Xiangya Hospital, Central South University, during December 2015 to December 2020. All the patients' baseline data, operation data, and follow-up data are collected. The study has been proved by ethics board of Second Xiangya Hospital, Central South University (#2021-150). All the patients have signed the consent to agree to supply their clinical and image data for medical research.

### Self-Radiopaque Physician-Modified Fenestration–Thoracic Endovascular Aortic Repair Procedure Details

#### The Inclusion Criteria and Exclusion Criteria on Patients Undergoing Self-Radiopaque Physician-Modified Fenestration–Thoracic Endovascular Aortic Repair

##### Inclusion Criteria

(1) The proximal landing zone [proximal lesion site to left subclavian artery (LSA)] for stent-graft is less than 15 mm; (2) lesions are located at Zones 1, 2, and 3 of the aorta; (3) disease group includes aortic dissection (AD)/penetrating aortic ulcer (PAU)/aneurysm/pseudoaneurysm and endoleak of prior TEVAR; (4) the left vertebral artery is identified as dominant by computed tomography angiography (CTA)/digital subtraction angiography (DSA) evaluation; (5) the left internal mammary artery is identified as the coronary bypass vessel; and (6) arterio-venous access for dialysis is located in left arm.

##### Exclusion Criteria

(1) Patients who cannot tolerate anesthesia according to anesthesiology assessment; (2) lesions involving the ascending aorta; (3) the diameter of aortic landing zone exceeds 44 mm; (4) severe calcification of the aortic arch; (5) branches on the aortic arch bear distal occlusion; and (6) patients suffering from malignant diseases whose anticipated survival duration is less than 2 years.

#### The Devices of Self-Radiopaque Physician-Modified Fenestration–Thoracic Endovascular Aortic Repair

**Figure 1:**
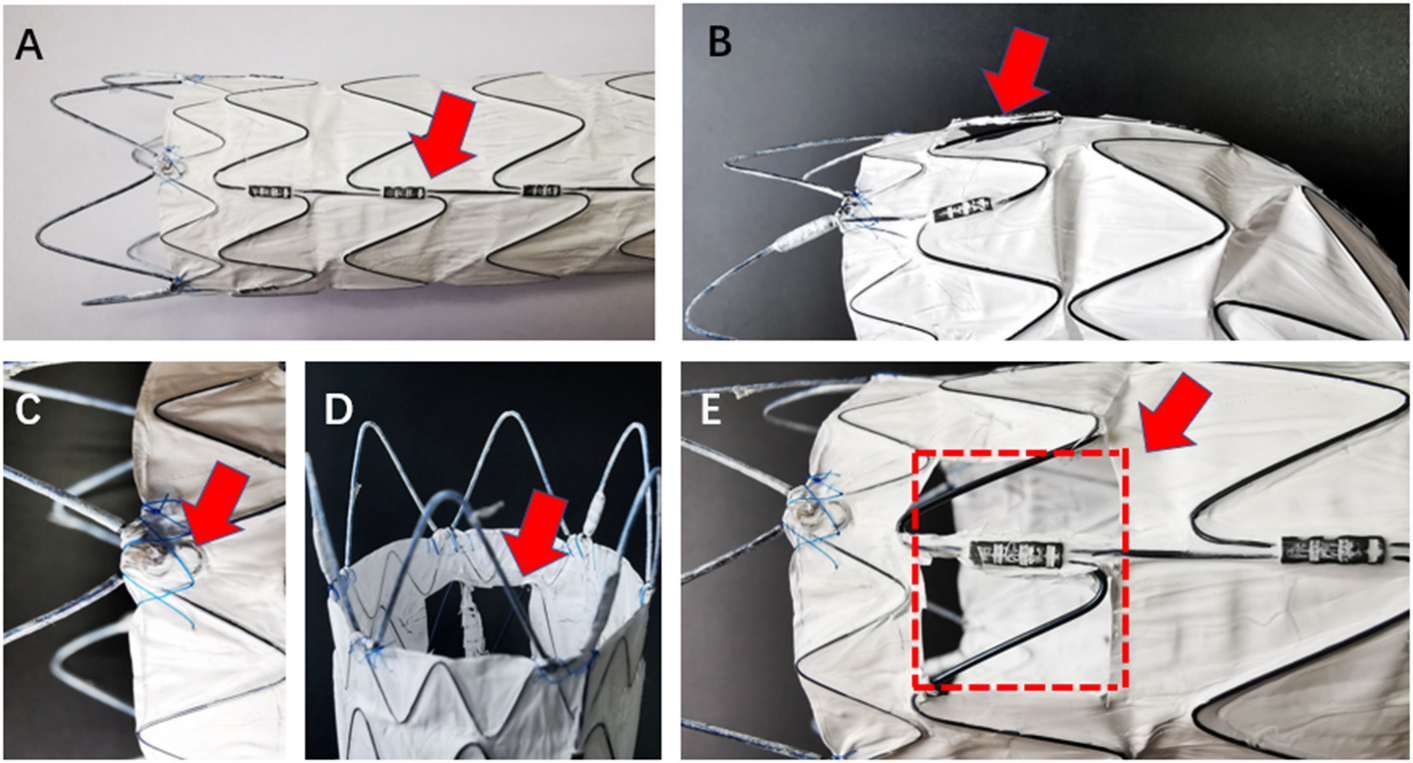
The model stent-graft (LifeTech Co., Shenzhen, China) shows radiopaque marker and formation after fenestration in different angle. **(A)** Red arrow shows the longitudinal strength strut, which is visible on radiation monitor; **(C)** red arrow shows the “∞” radiopaque marker at the proximal site of the membrane edge; **(B,D,E)** fenestration in multiple angles. Red arrows show the complete fenestration. Red dot-line shows the area and position of the fenestration.

**Figure 2:**
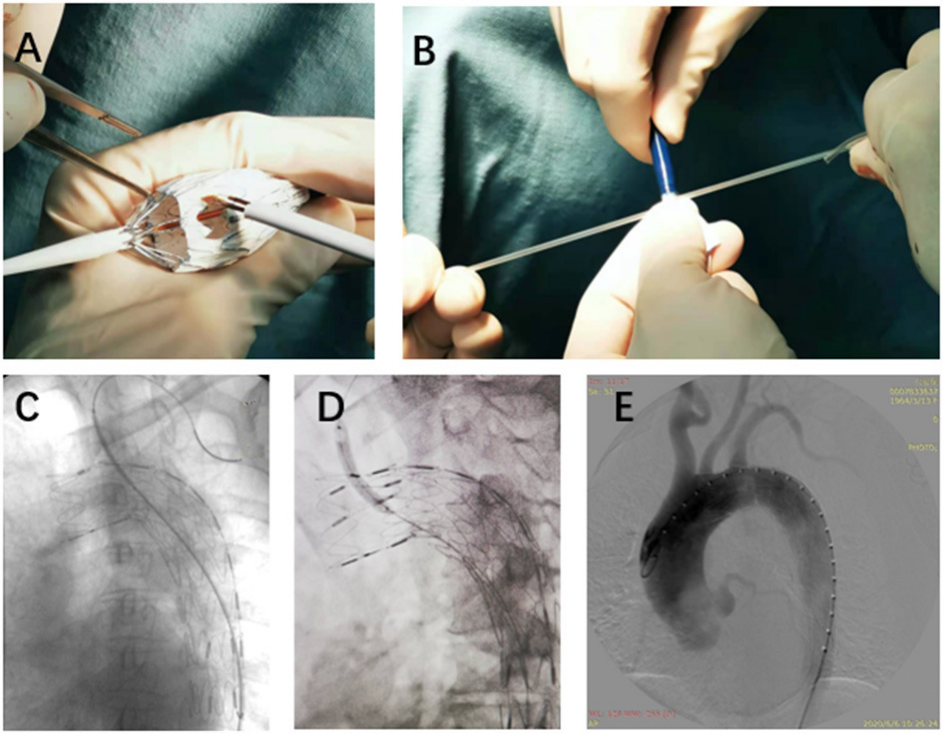
The details of SF-TEVAR procedures. **(A)** On-table fenestration process. **(B)** Reloading the stent-graft back into the outer sheath after the fenestration. **(C)** After delivery of the fenestrated stent-graft, a wire connected the aorta and the LSA (from the same access in femoral artery), and an expandable balloon stent was placed through the fenestration. **(D)** Inflation of the balloon and deployment of the stent. **(E)** DSA after the SF-TEVAR procedures. SF, self-radiopaque physician-modified fenestration; TEVAR, thoracic endovascular aortic repair; LSA, left subclavian artery; DSA, digital subtract angiography.

(1) Ankura aortic stent-graft (LifeTech Co., Shenzhen, China) is the brand of stent-graft for SF-TEVAR because its special radiopaque markers provide reliable guidance for fenestration alignment (Figure 1); (2) Express LD stent (Boston Scientific Co., Marlborough, MA, USA) and Viabahn endograft (W.L. Gore & Associates, Flagstaff, AZ, USA) are used for bridging the aorta and LSA across the fenestration; (3) vertebral artery catheter (C2) (Cordis Corporation, Miami Lakes, FL, USA) is used to cannulate the aorta and LSA and make the guidewire exchange in position; (4) gold marker pigtail catheter (Cook Group Incorporated, Bloomington, IN, USA); (5) Lunderquist superstiff guidewire (William COOK Europe, Bjaeverskov, Denmark); (6) 0.035 super slippery guidewire (Terumo, Hanoi City, Vietnam); (7) Fluency Plus self-expanding covered stent (Angiomed GmbH & Co., Karlsruhe, Germany) is used as a bailout stent for the chimney technique; and (8) electro-coagulation instrument (Bovie Medical Corporation, Clearwater, FL, USA) is used for removing the membrane on the aortic endograft.

#### Self-Radiopaque Physician-Modified Fenestration–Thoracic Endovascular Aortic Repair Procedure Protocol

**Figure 3:**
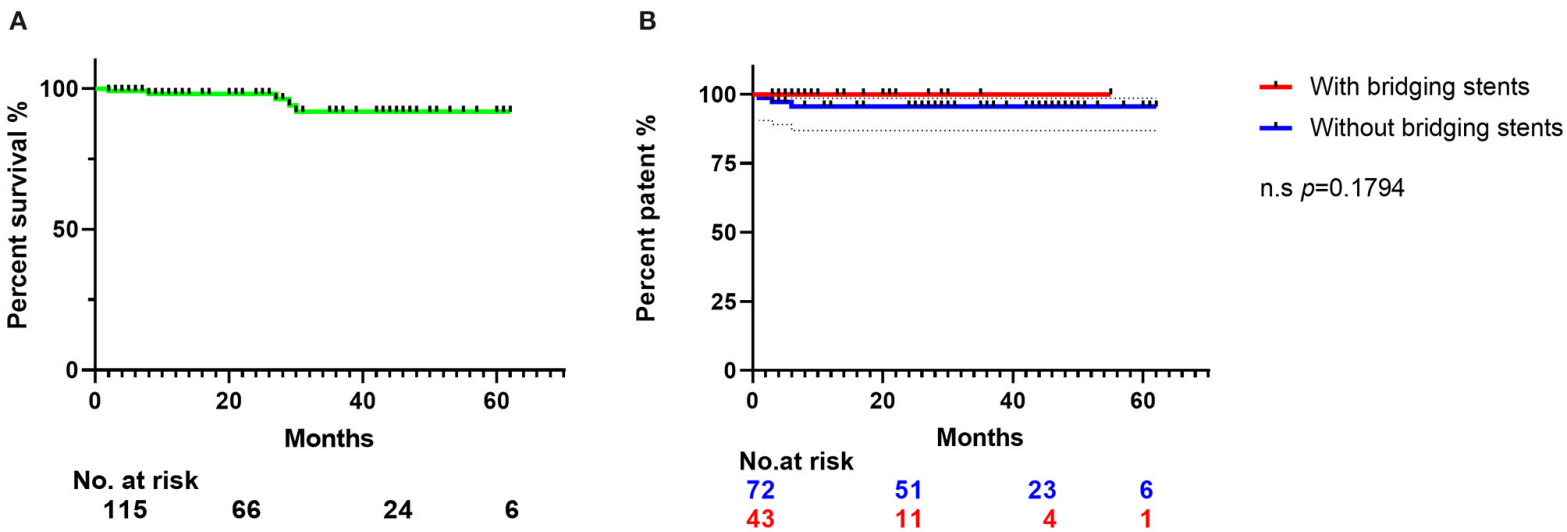
The Kaplan–Meier curve shows the survival rates of the 115 patients' follow-up data. **(A)** The Kaplan–Meier curve of the patients' survival rates in 23 ± 18 months' follow-up. **(B)** Patent rates of branch arteries preserved by fenestration. Blue line represents the SF-TEVAR without bridging stent, and red line represents the SF-TEVAR with bridging stent. There is no significant difference between the patent rate of two groups (*p* = 0.1794). CTA, computed tomography angiography; LSA, left subclavian artery; SF, self-radiopaque physician-modified fenestration; TEVAR, thoracic endovascular aortic repair; PAU, penetrating aortic ulcer; LCA, left carotid artery.

All procedures were accomplished in hybrid theatre. The patients were under general anesthesia with intubation during the operation. Unilateral femoral artery access was dissected or pre-positioned with two vascular sealing instruments (Abbott Vascular, Santa Rosa, CA, USA). Surgical sterilization of puncture or incision sites of the intentionally preserved the arteries (e.g., for LSA, prepare the cubital area for the branchial artery; for the left carotid artery (LCA), prepare the cervical area for the distal common carotid artery). The specific procedures are as follows: (1) perform pre-procedural angiography. The direction of X-ray tube is vertical to the dimension of aortic arch; (2) partially release the outer sheath of the aortic stent-graft and expose 4–5 cm of the stent-graft. Electro-coagulation instrument is used to remove part of the membrane. The position of the fenestration is on the rear end of the “∞” radiopaque marker. Another radiopaque marker “strut” is kept in the central line of the fenestration. The distance between “∞” and proximal edge of fenestration is determinate by proximal landing zone and is individually dependent (Figures 1, 2); (3) reload the fenestrated stent-graft back into the sheath and flush by heparin saline; (4) apply the stent-graft to aortic arch along with the guidewire through femoral access; (5) carefully rotate the stent-graft for accurate alignment, until the “∞” radiopaque marker shows as “–” on the monitor and the strut is attached to the great curvature of aortic arch. Then fully deploy the stent-graft; (6) perform DSA to ensure the patency of the intentionally preserved branch artery; (7) cross the vertebral catheter C2 from the femoral artery to the fenestration, and exchange the guidewire connecting the branch and the aorta (Figure 2); (8) deliver the bare/covered stent as the bridging stent through the fenestration by wire, and keep approximately 1–1.5 cm in the aorta and the remaining part in the branch artery (Figure 2 ); (9) perform another angiography to validate the correct position of aortic endograft and bridging stent, and check if endoleak occurs (Figure 2); and (10) close the incision of vascular access by surgical suturing or sealing instruments.

### Data Collection, Follow-Up, and Statistics

The patients' baseline data and clinical characteristics were collected from electronic history system of Second Xiangya Hospital. The complete data of procedure time, operation time, contrast volume, stent-graft, and bridging stent were collected for further analysis. Serial CTA follow-up was required at 1, 6, and 12 months and then once every year after the procedures. Follow-up primary endpoints are (1) patients died and (2) patients need re-intervention in relation to last the TEVAR procedure. Secondary endpoints are (1) major adverse cardiovascular events; (2) Stanford Type A dissection diagnosed after the TEVAR procedure; and (3) branch kept by SF occluded after procedure.

The numerical data are shown as average ± standard deviation. Accumulated survival analysis was performed with the Kaplan–Meier method. Statistical analysis and graphing were performed with GraphPad Prism (8.0 GraphPad Software, San Diego, CA, USA).

## Results

**Figure 4:**
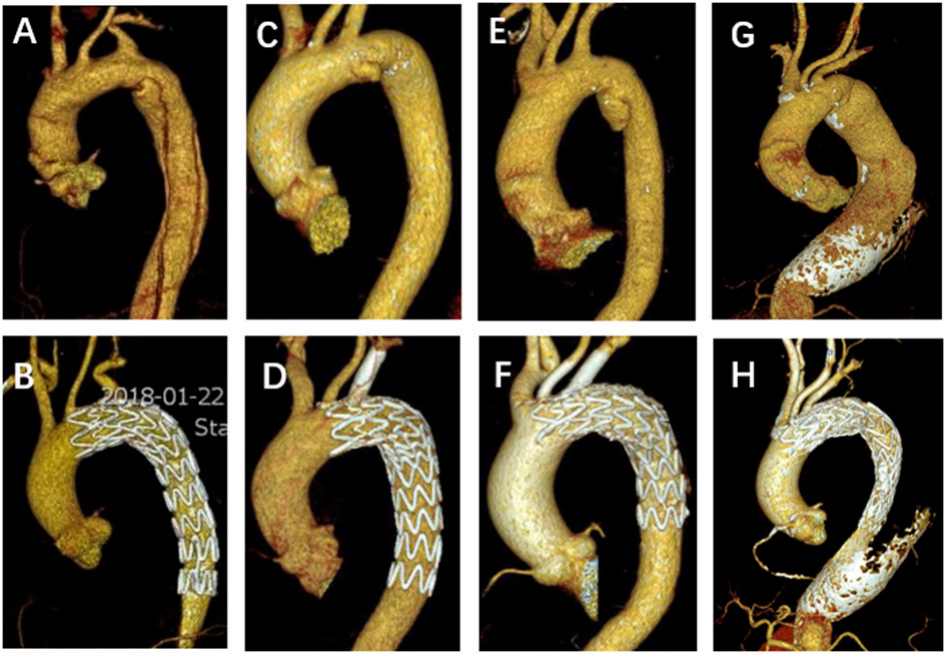
Representative pictures of preoperative and follow-up CTA. **(A,B)** A non-bridging stent for LSA SF-TEVAR in a typical aortic dissection patient. **(C,D)** A single fenestration for LSA and one bridging stent implantation in a PAU patient. **(E,F)** Double-bridging stents for both LSA and LCA SF-TEVAR in a PAU patient. **(G,H)** A large fenestration for triple aortic arch branches without bridging stents in a case of arch and descending aortic aneurysm. CTA, computed tomography angiography; LSA, left subclavian artery; SF, self-radiopaque physician-modified fenestration; TEVAR, thoracic endovascular aortic repair; PAU, penetrating aortic ulcer; LCA, left carotid artery.

This is a single-center retrospective cohort study of 125 patients who underwent SF-TEVAR, including 107 male (85.6%) and 18 female patients (14.4%). The mean age (years) is 56.9 ± 10.9. The types of aortic arch lesion in this study include acute AD (63 cases, 50.4%), PAU (38 cases, 30.4%), aortic arch aneurysm (15 cases, 12.0%), aortic pseudoaneurysm (two cases, 1.6%), intramural hematoma (five cases, 4.0%), and type I endoleak after previous TEVAR (two cases, 1.6%). The general information of clinical characteristics is listed in Table 1. There are two cases where fenestrations were not accurately aligned to the branch orifice. In one of the patients, bail-out chimney stent was implanted. For the other, the misalignment was corrected by multi-balloon inflation to readjust the fenestration location. In general, the technique success rate is 98.4%. There were total of 132 aortic endografts implanted during this study including 54 bridging stents in the branch arteries, where 44 bridging stents were applied in LSA, eight in the LCA, and two in the innominate artery (IA). Of interest, there were two cases of triple-branch fenestration to keep all the arch branches patent (1.6%). Double-branch fenestrations were also performed for the LCA and LSA in six cases (4.8%). One hundred seventeen single-branch fenestrations were performed for LSA (93.6%) (Table 2). Statistically, the total operation time is 64.6 ± 19.3 min, X-ray exposure time (from the first angiography to the last angiography) is 25.6 ± 14.3 min, contrast volume is 82.2 ± 22.6 ml, and the 30-day mortality is 0. However, there were five patients died during follow-up due to other complications/events (Table 3). Type I endoleak was found in seven patients (5.6%), and the occlusion of the branch artery occurred in three patients (2.4%). The overall patent rate of the branch artery is 97.6%. There is no significant difference on branch artery patent rate between bridging stent group and non-bridging stent group (*p* = 0.1794) (Figure 3). In the follow-up CT pictures, we found that the aorta remodeling is satisfactory. Representative CT follow-up data are shown in Figure 4. There is no bridging stent under condition of migration or collapse noticed in the CT data.

**Table 1:** General information and characteristics of the patients.

Age	56.9 ± 10.9 yrs
Duration of symptoms onset to admission	31.2 ± 99.9 day
Duration of admission to TEVAR	8.0 ± 4.9 day
**Gender**	
MaleFemale	107 (85.6%) 18 (14.4%)
**Comorbidities**	
Hypertension Coronary artery diseases Hyperlipemia Diabetes Mellitus Chronic kidney diseases Cerebral infarction/bleeding Chronic heart failure COPD	98 (78.4%) 12 (9.6%) 20 (16.0%) 12 (9.6%) 9 (7.2%) 8 (6.4%) 1 (0.8%) 1 (0.8%)
**Clinical manifestations**	
Chest pain Back pain Abdominal pain Others No symptoms	73 (58.4%) 62 (49.6%) 13 (10.4%) 6 (4.8%) 29 (23.3%)
**Types of aortic pathology**	
Acute aortic dissection Chronic aortic dissection Penetrating aortic ulcer Intramural hematoma Type I endoleak after TEVAR	60 (48.0%) 3 (2.4%) 38 (30.4%) 5 (4.0%) 1 (0.8%)
**Aortic anatomic abnormalities**	
Bovine aortic arch Left vertebral artery from arch Aberrant right subclavian artery	11 (8.8%) 10 (8.0%) 2 (1.6%)
**Pathologies reach zones**	
Zone 0 Zone 1 Zone 2 Zone 3	1 (0.8%) 2 (1.6%) 51 (40.8%) 72 (57.6%)

*COPD, chronic obstructive pulmonary disease; TEVAR, thoracic endovascular aortic repair.*

*The age is shown as average ± standard deviation, and category data was shown as number (percentage)*.

**Table 2:** Information in SF-TEVAR procedures and peri-operative complications (within 30 days of SF-TEVAR).

Aortic stent-graft brand	Number (%)
Lifetech-Ankura® I/II	129 (97.7)^*^
Reconstructions of the aortic branches type	Number of branch (%)
LSA LCA IA Mono-fenestration Dual-fenestration Triple-fenestration	125 (100) 8 (6.4) 2 (0.8) 117 (93.6) 6 (4.8) 2 (1.6)
Bridging stent/covered stent distribution	Number of stent (%)
LSA (no stent/ bare stent/ covered stent) LCA (no stent/ bare stent/ covered stent) IA (no stent/ bare stent/ covered stent)	81 (64.8)/35 (28.0)/9 (7.2) 117 (93.6)/3 (2.4)/5 (4) 123 (98.4)/0 (0)/ 2 (1.6)
Complications:	Number of patient (%)
Type I endoleak Pneumonia/pleural effusion SCI Bridging stent occlusion MACE Stroke Retrograde type A dissection SINE Stent-graft infection	7 (5.6) 3 (2.4) 1 (0.8) 1 (0.8) 0 (0) 0 (0) 0 (0) 0 (0) 0 (0)

*LSA, left subclavian artery; LCA, left common carotid artery; IA, innominate artery; SCI, spinal cord ischemia; MACE, major adverse cardiovascular events; SINE, stent-graft induced new entry.*

* *The total number of aortic stent-grafts is 132. The other brand of stent-grafts were used in distal site without PMF*.

**Table 3:** Death causes and complications in follow-up (after discharge).

**Death causes**	**Time from discharge (months)**
#1 Pts Myocardial infarction	30
#2 Pts Cerebral artery aneurysm rupture	29
#3 Pts Vehicle accident	27
#4 Pts Major digestive bleeding	8
#5 Pts Falling down from bed	1
**Complications in follow-up**	Number of patient (%)^*^
Late endoleak MACE Stroke Retrograde type A dissection SINE SCI Stent-graft infection Bridging stent occlusion	0 (0) 1 (0.9) 0 (0) 1 (0.9) 1 (0.9) 0 (0) 0 (0) 2 (1.7)

* *The total number of follow-up patients is 115 and there are 10 patients that lose contact. Abbreviations: MACE, major adverse cardiovascular events; SINE, stent-graft induced new entry; SCI, spinal cord ischemia*.

## Discussions

TEVAR has been rising as the first-line method to repair Stanford B AD due to its advantages of minimal invasion and less complication (12). When the pathologic lesions involve the aortic arch, it is likely that physicians choose procedures such as a hybrid operation (13, 14). Although the hybrid procedures already realize the purpose of less trauma, it is preferable to conduct the repair with more endovascular and less open surgery. Recently, the chimney technique and fenestration technique are the most frequently used “off-the-shelf” methods by specialists who treat the aortic arch pathologies (15, 16). Moreover, the PMF procedure is more suitable for aortic arch anatomy, with even less rate of endoleak compared with the chimney technique (10, 17, 18). In this section, we discuss the advantages and our perspectives on SF-TEVAR in the repair of aortic arch lesions.

### Radiopaque Marker in Stent-Graft Improves Alignment Accuracy

The stent-graft used in this study is Chinese brand LifeTech-Ankura®, where the specially designed radiopaque marker on the stent helps physician to locate fenestration and align puncture to branch arteries in an easy and efficient way (Figure 1). On the one hand, there is a “∞” marker located at the border between bare stent and membrane. In practice, the distance between the “∞” marker and the proximal border of the fenestration equals to the distance between intentionally preserved the branch artery and its proximal branch artery orifice. On the other hand, there is also another important radiopaque marker—the strength strut, a longitudinal strut located on the one side of the stent-graft. When performing TEVAR by this stent-graft, the strut was always instructed to be put in the great curvature of the aortic arch. The strut will help to reveal any spiral movement of the stent-graft. During the fenestration procedure, we always position the strut at the middle longitudinal axis. When aligning the fenestration to the branch artery, the “∞” marker and strut, which are shown as a dot and a line, respectively, will help physicians to form a spatial conception of the stent-graft. If performing fenestration on other brands of stent-graft, it is possible to suture radiopaque markers such as wires to serve this purpose. However, it would be very time-consuming. Besides radiopaque markers, the radiation angle is also important for locating and alignment. It is theoretically more accurate to make alignment by exposing the radiation as vertical to the virtual dimension of the aorta. Based on our experience so far, the radiation angle of left anterior oblique (LAO) of 46° works in most situations. In our early experience of PMF, we tended to make a larger fenestration (15 mm ×15 mm) in order to increase the puncture alignment rate. However, as the other side of the coin, a large fenestration may increase the rate of endoleak (15, 19). In recent years, we have achieved a more accurate alignment rate by SF-TEVAR technique and have used more bridging stents. Therefore, the fenestration size was able to be reduced, which brings better outcome with less endoleak rate (20).

Although the alignment rate of SF-TEVAR is satisfactory, chances of certain failure still exist. The routine bail-out method is to implant a covered stent by the chimney technique. The other alternative method is to use a guidewire to first “find” a tiny fissure existing in the misalignment fenestration area and perform cannulation, followed by serial and gradual balloon dilation to rescue the fenestration position right to the branch artery, slowly and eventually deploying bridging stent, which avoids the relatively high rate of type I endoleak by chimney and corrects the misalignment of fenestration ideally.

### Placing Bridging Stent Comfortably and Less Invasively

Another significant advantage of SF-TEVAR is easy implanting of the bridging stents. First, we use the same access for aortic stent-grafts and do not require extra access from the distal LSA or LCA in most situations. In our procedure, a C2 vertebral catheter from femoral access cannulates the aorta and LSA/LCA through the fenestration. After exchange of guidewire, a bare stent or covered stent will bridge the fenestration, which makes the whole morphology of stent-grafts as similar to normal anatomy. Although the pull-out force of the bridging stent is of concern for experts who perform a similar procedure (21, 22), there are no adverse events found, such as bridging stent fracture or dislocation, in our follow-up results. Even better, the bridging stent will decrease the endoleak rate (10, 23, 24). It is due to these two advantages from bridging stent that another percutaneous puncture is not needed in our experience. Generally, SF-TEVAR procedure require less time than other techniques, and it cuts down the time of general anesthesia, which benefits aged patients from short anesthesia resuscitation (25) and also explains the low occurrence of perioperative major adverse cardiovascular events (MACEs) and other complications.

### About the Endoleak

It is unavoidable to notice the endoleak in TEVAR procedures. When performing TEVAR especially in the aortic arch, decreasing endoleak rate is essential for a procedural success. Ahmed et al. reported that the endoleak rate of the chimney technique is around 9.4% (26), which is similar to the rate of the authors' center (4, 5). Also, some literatures report that the endoleak rate of *in situ* fenestration is even higher (27, 28). In our study, the endoleak events have a low rate of 5.6%, and it only persists in two patients who received a large fenestration stent for all of the three artery branches. In our experience, most endoleak happens in SF-TEVAR with multi-branch fenestration, which is not difficult to interpret since large and multiple fenestration brings higher chance of gap and fissure (29). However, the instant endoleak does not necessarily persist for a prolonged time. In our study, there are five perioperative endoleak cases that disappeared spontaneously in the 6-month follow-up. We believe that bridging stent implantation and strict hypertension control contributed to the spontaneous disappearance of minor endoleak, and it is worthy to use covered stent as bridging stent implantation in the future because it may bring better results with decreased endoleak rate.

### Complications and Survival Situation

During long-term follow-up, five patients died from non-aortic-related causes. This result at least proves that the safety and efficacy of SF-TEVAR in the midterm and long term is acceptable. For the perioperative complications except endoleak, pulmonary infection and spinal cord ischemia (SCI) rate is low, which is reasonable since LSA is preserved, which perfuses posterior and anterior spinal artery systems (30–32). Specifically, there was one patient with transient paraplegia, but he recovered spontaneously in 1 h without walking or defecation disabilities. His paraplegia recovery was so quick that we had prepared the spinal drainage but became unnecessary. One case of branch artery occlusion in the perioperative period occurred, although all patients received aspirin 100 mg once per day. The patient was from the non-bridging stent group, but the case did not make a statistical difference on complication between non-bridging and bridging stent groups, which indicates that a larger sample group may be needed to discover the potential difference. One patient developed retrograde type A dissection probably due to his extremely poorly controlled hypertension. One patient underwent the SINE (stent-related complications), which is also due to persisting hypertension. We suppose that it serves as a good reminder that long-term hypertension management is of great importance to patients who received TEVAR including SF-TEVAR.

When we develop techniques with more “endo” device to minimize the patients' risk and trauma, the main goal is to bring better clinical outcome and life quality. SF-TEVAR is favorably used to save patients from possible trauma of thoracotomy, sternotomy, subsequent aortic cross clamping, and bypass establishment. Extra-anatomic debranching not only requires an incision but also has its own particular risks to obtain vertebral and mammary artery preservation. However, both “endo” and “open” will still have their position in treating aortic pathologies due to their own special merits but also complication characteristics **(****33****)**. Endovascular devices and techniques will continue to improve and evolve and will be the procedure of choice in the future when these developments are fully realized.

### Limitation of This Study

The limitation of this study is that is a single-arm, retrospective study in a single center. We would like to check the SF technique on the patient whose LSA has been covered by TEVAR directly. However, since we developed the SF technique, very few patients' LSAs had been covered, and we may need to wait for more patients in this arm. Further, we may organize a multiple-center comparison of this technique in the future. Another limitation is bridging stent selection and comparison. As the covered-balloon expandable stent has been introduced in China, we shall generate some data for their use compared with bare stent as bridging stent.

## Conclusions

The SF-TEVAR technique, which utilizes the radiopaque marker in stent-graft as an indication for PMF in TEVAR, seems a likely safe, effective, and efficient procedure, which brings acceptable survival rate and branch artery patency rate. SF-TEVAR serves as a progressive alternative method to keep the branch artery patent in aortic arch endovascular reconstruction.

## Data Availability Statement

The raw data supporting the conclusions of this article will be made available by the authors, without undue reservation.

## Ethics Statement

This study is approved by the Ethics Committee of the Secondary Xiangya Hospital, Central South University. The patients/participants provided their written informed consent to participate in this study. Written informed consent was obtained from the individual(s) for the publication of any potentially identifiable images or data included in this article.

## Author Contributions

CS conceived the idea and designed the whole article. XL collected the data and wrote the article. QL, HH, ML, LW, and JL collected the data. DL and MD performed the statistical work and wrote the article. All authors contributed to the article and approved the submitted version.

## Conflict of Interest

The authors declare that the research was conducted in the absence of any commercial or financial relationships that could be construed as a potential conflict of interest.

## Publisher's Note

All claims expressed in this article are solely those of the authors and do not necessarily represent those of their affiliated organizations, or those of the publisher, the editors and the reviewers. Any product that may be evaluated in this article, or claim that may be made by its manufacturer, is not guaranteed or endorsed by the publisher.

## Abbreviations

3D: three dimensional
AD: aortic dissection
CTA: computed tomography angiography
DSA: digital subtraction angiography
IA: innominate artery
LAO: left anterior oblique
LCA: left carotid artery
LSA: left subclavian artery
MACE: major adverse cardiovascular event
PAU: penetrating aortic ulcer
PMF: physician-modified fenestration
SCI: spinal cord ischemia
SF: self-radiopaque physician-modified fenestration
SINE: stent-graft induced new entry
TEVAR: thoracic endovascular aortic repair.
